# Static and dynamic analyses of free-hinged-hinged-hinged-free beam in non-homogeneous gravitational field: application to gravity gradiometry

**DOI:** 10.1038/s41598-022-11232-6

**Published:** 2022-05-04

**Authors:** Alexey V. Veryaskin, Thomas J. Meyer

**Affiliations:** 1 Trinity Research Labs, School of Physics, Mathematics and Computing, University of Western Australia, 35 Stirling Highway, Crawley, WA 6009 Australia; 2 Quantum Technologies and Dark Matter Research Laboratory (QDM Lab), Department of Physics, University of Western Australia, 35 Stirling Highway, Crawley, WA 6009 Australia; 3 Lockheed Martin RMS – Gravity Systems, 2221 Niagara Falls Boulevard, Niagara Falls, NY 14304 USA

**Keywords:** Mechanical engineering, Applied physics, Techniques and instrumentation

## Abstract

The first analytical evaluation of a free-hinged-hinged-hinged-free beam proposed for use as the primary sensing element of a new gravity gradiometer is presented. Results of the evaluation obtained in quadratures are applied to the beam’s structure, including locating the hinges that form the beam’s boundary conditions allowing only free rotations around its nodal axes. These are deliberately chosen to minimize the beam’s symmetric free ends deflections under the uniform body loading of gravity while simultaneously permitting the beam’s maximum possible mirror-symmetric free ends deflections owing to a gravity gradient distributed along its length. The flexible triple-hinged beam deformation from its nominal unloaded geometry is naturally elastically coupled throughout, including free ends, allowing synchronized mechanical displacement measurements at any deflection point. Some methods of manufacturing such sensing elements and their respective error mechanisms are also discussed and presented for the first time.

## Introduction

Distributed flexible mechanical objects such as cantilevers^1^, flexures used in MEMS^2–4^ and strings/ribbons^5^ have been in use for quite a long time as primary sensing elements (test masses) for measuring gravitational acceleration and its spatial derivatives (gravity gradients). By measuring the latter, one could get, for example, valuable information about buried mineral deposits, hidden underground voids, tunnels and bunkers, and use the fine Earth’s gravity data for passive, not jammable, strategic submarine navigation^6^. The difference between the primary sensing elements in the form of distributed flexible test masses (elongated beams and ribbons) and compact solid test masses (spheres, cylinders and the like) is that in the latter case the test masses are responsive to the local force of gravity applied to centres-of-mass, while in the former case they can be more receptive to the force-per-unit-length that is, by definition, directly proportional to gravity gradients. The distributed test masses allow for the construction of a continuous beam type gravity gradiometer where only one sensing element is needed for measuring a gravity gradient along its length^7^. If compact test masses are used, a minimum of two of them are needed to measure the difference in the force of gravity acting upon them. In the latter case one needs to maintain a fixed spatial separation of the test masses or “baseline” which effectively determines sensitivity and size of the resulting gradiometer instrument. The stand-alone test masses and the corresponding sensing means, that translate their motion into a measurable physical quantity, must be matched with an unprecedented accuracy in order to meet a state-of-the-art measurement capability (typically within a few parts per billion for requisite ultra-precision) which, in turn, must be provided over the whole gradiometer run time, i.e. must be stable within reasonably long time intervals.

**Figure 1:**
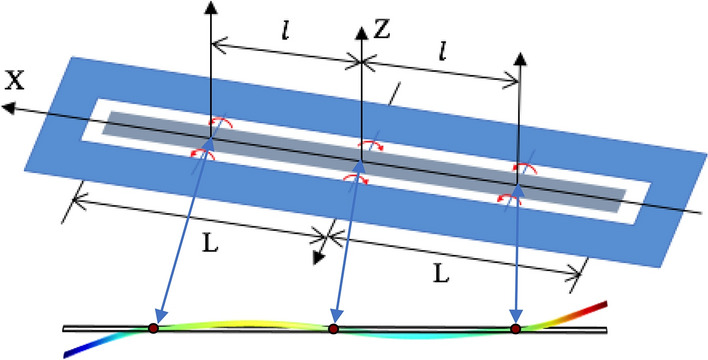
A mirror-symmetric profile of an elongated thin metal beam (the gray colored ribbon in the middle of a solid blue reference frame) which is hinged at three equally separated axes and having overhanging free ends. Such deformation profile also shown is the result of a linearly distributed force-per-unit-length, representing a pure gravity gradient load.

A primary sensing element having a single sensitivity axis is shown in Fig. 1 below. It comprises an elongated thin metal beam (ribbon or foil) which is hinged at three equally separated axes and having overhanging free ends^8^. In beam theory it might be called a free-hinged-hinged-hinged-free beam. The hinges are connected to so-called zero-force frame of reference providing free beam’s rotational motion around their axes.

The deflection of the beam’s free ends with respect to its unperturbed position can be measured by an appropriate mechanical-displacement-to-voltage conversion technique such as a moving plate capacitive transducer^9,10^. The locations of the hinges are sought to simultaneously minimize the beam’s symmetric, with respect to the central axis, free ends deflection under the uniform force distribution while permitting the beam’s maximum possible mirror-symmetric deflection due to a gravity gradient along its length. The beam’s free-hinged-hinged-hinged-free structure is heavily over-constrained, and to the best of the authors’ knowledge has not been analytically evaluated before in open publications. For the first time a static and dynamic analyses, including error factors, of such over-constrained beam loaded by a non-uniform force distribution is presented below.

## Theoretical framework

There are well known theoretical frameworks that have been widely used to analyse the general transverse motion of elongated flexible beams under different boundary conditions, which are Euler–Bernoulli–Lagrange theory^11^ and Rayleigh-Timoshenko theory^12–14^. In the static approximation, they coalesce to the same framework where the transverse displacement *Z* of every point of a beam under investigation and its bending slope *θ* are described by the following equations

1 $$\frac{EI}{\eta }\frac{{d^{4} Z}}{{dx^{4} }} = g_{z} \left( {x,z} \right) \cong g_{z} \left( 0 \right) + {\Gamma }_{zx} x$$

2 $$\frac{EI}{\eta }\frac{{d^{4} \theta }}{{dx^{4} }} = \frac{d}{dx}g_{z} \left( {x,z} \right) \cong {\Gamma }_{zx}$$

3 $$\theta = \frac{dZ}{{dx}}$$

where *E* is the beam’s Young modulus of elasticity, *I* is the beam’s area moment of inertia, *η* is the beam’s mass per unit length, *g*_*z*_ is normal to the beam surface gravitational acceleration vector component and Γ_zx_ is a gravity gradient tensor component along the beam’s length

4 $${\Gamma }_{zx} = \frac{{g_{z} \left( L \right) - g_{z} \left( { - L} \right)}}{2L}$$

The beam’s area moment of inertia *I* is described by the following equation^15,16^

5 $$I = \frac{1}{12}\frac{{bd^{3} }}{{1 - \sigma^{2} }}$$

where *b* and *d* are the width (base) and thickness of the beam accordingly, *σ* is Poisson ratio.

In Eqs. (1) and (2) an extra term $\Gamma_{zz} z$ has been ignored in the first order series expansion of the gravitational acceleration component *g*_*z*_*(x,z)* over coordinates *x* and *z*, where $\Gamma_{zz}$ is the vertical gravity gradient component. This term, if left there, would introduce so-called gravitational spring, that can be either positive or negative, since *Z* = *z* represents the beam’s transverse displacement variable. Including this term leads to unnecessary complication of the quasi-static analysis as the corresponding corrections are the second order of magnitude. Also, this term is a symmetric one with respect to $x \to - x$ coordinate transfer and therefore can not modify the beam’s deflection under the gradient term $\Gamma_{zx}$.

For the free-hinged-hinged-hinged-free beam the following boundary conditions are applied to Eqs. (1) and (2)

6 $$Z\left( { - l} \right) = Z\left( 0 \right) = Z\left( l \right) = 0$$

7 $$\frac{{d^{2} \theta }}{{dx^{2} }}|_{x = - L} = \frac{{d^{2} \theta }}{{dx^{2} }}|_{x = L} = 0$$

8 $$\frac{d\theta }{{dx}}|_{x = - l} + \frac{d\theta }{{dx}}|_{x = l} + 4\frac{d\theta }{{dx}}|_{x = 0} = \frac{1}{2}\eta l^{2} g_{z} \left( 0 \right)$$

Equation (8) above is the manifestation of the “three moment” theorem that is applicable to any flexible beam’s adjoint three-span sections^8^. In the error-free model above, it is assumed that the hinges are aligned along the same XOY plane and separated by exactly the same spans. Also, the beam’s free ends are separated by exactly the same distance from the middle point x = 0 of the static coordinate system XYZ where the X-axis coincides with the unperturbed position of the beam. It is also assumed that the beam’s length (2L) is much larger compared to its width (b) and thickness (h) so the one-dimensional problem above is well-justified^17^.

The Eqs. (1) and (2) cannot be solved in quadratures for the whole length of the beam due to the number of boundary conditions vastly exceeding the order of the linear differential equations. However, they can be solved for each of the four spans along the beam’s length provided the continuity of the solutions at the nodal points is preserved^18^.

9 $$\theta \left( {l + \varepsilon } \right) = \theta \left( {l - \varepsilon } \right), \varepsilon \to 0$$

10 $$\theta \left( { - l - \varepsilon } \right) = \theta \left( { - l + \varepsilon } \right), \epsilon \to 0$$

11 $$\theta \left( {0 + \varepsilon } \right) = \theta \left( {0 - \varepsilon } \right), \epsilon \to 0$$

12 $$\frac{d\theta }{{dx}}|_{l + \varepsilon } = \frac{d\theta }{{dx}}|_{l - \varepsilon } , \varepsilon \to 0$$

13 $$\frac{d\theta }{{dx}}|_{ - l - \varepsilon} = \frac{d\theta }{{dx}}|_{ - l + \varepsilon } , \varepsilon \to 0$$

14 $$\frac{d\theta }{{dx}}|_{0 + \varepsilon} = \frac{d\theta }{{dx}}|_{0 - \varepsilon } , \varepsilon \to 0$$

It is worth noting that the bending moments *EId* $\theta$*/dx* at the beam’s free ends do not vanish in the presence of the force gradient along the beam’s length. For the sake of simplicity, the details of solving Eqs. (1) and (2) for four adjoint spans independently are omitted here and the final results are shown below

$$l \le x \le L$$

15 $$\begin{aligned} Z\left( {\text{x}} \right) & = - {\Gamma }_{zx} {\upeta }\frac{{\left( {l - x} \right)\left( {10l^{4} - 27l^{3} x - 30L^{2} x^{2} + 3x^{4} - l^{2} \left( {30L^{2} - 3x^{2} } \right) + 3l\left( {20L^{2} x + x^{3} } \right)} \right)}}{360EI} \\ & \,\,\,\,\,\,\,\,\, + g_{z} \left( 0 \right){\upeta }\frac{{\left( {l - x} \right)\left( {l^{3} - 2l^{2} \left( {2L + x} \right) + l\left( {6L^{2} + 8Lx - 2x^{2} } \right) - 2x\left( {6L^{2} - 4Lx + x^{2} } \right)} \right)}}{48EI} \\ \end{aligned}$$

16 $$\begin{aligned} \theta \left( x \right) & = {\Gamma }_{zx} {\upeta }\frac{{37l^{4} - 90l^{2} L^{2} - 60l^{3} x + 180lL^{2} x - 15x^{2} \left( {6L^{2} - x^{2} } \right)}}{360EI} \\ & \,\,\,\, - g_{z} \left( 0 \right){\upeta }\frac{{3l^{3} - 12l^{2} L + 18lL^{2} - 8x\left( {3L^{2} - 3Lx + x^{2} } \right)}}{48EI} \\ \end{aligned}$$

$$0 \le x \le l$$

17 $$Z\left( {\text{x}} \right) = {\Gamma }_{zx} {\upeta }\frac{{x\left( {7l^{4} - 10l^{2} x^{2} + 3x^{4} } \right)}}{360EI} - g_{z} \left( 0 \right){\upeta }\frac{{\left( {l - x} \right)x^{2} \left( {3l^{2} + 6L^{2} - 2l\left( {6L - x} \right)} \right)}}{48EIl}$$

18 $$\theta \left( x \right) = {\Gamma }_{zx} {\upeta }\frac{{7l^{4} - 30l^{2} x^{2} + 15x^{4} }}{360EI} - g_{z} \left( 0 \right){\upeta }\frac{{x\left( {6l^{3} - 18L^{2} x - 3l^{2} \left( {8L + x} \right) + 4l\left( {3L^{2} + 9Lx - 2x^{2} } \right)} \right)}}{48EIl}$$

$$- L \le x \le - l$$

19 $$\begin{aligned} Z\left( {\text{x}} \right) & = {\Gamma }_{zx} {\upeta }\frac{{\left( {l + x} \right)\left( {10l^{4} + 27l^{3} x - 30L^{2} x^{2} + 3x^{4} - l^{2} \left( {30L^{2} - 3x^{2} } \right) - 3l\left( {20L^{2} x + x^{3} } \right)} \right)}}{360EI} \\ & \,\,\,\,\, + g_{z} \left( 0 \right){\upeta }\frac{{\left( {l + x} \right)\left( {l^{3} - 2l^{2} \left( {2L - x} \right) + l\left( {6L^{2} - 8Lx - 2x^{2} } \right) + 2x\left( {6L^{2} + 4Lx + x^{2} } \right)} \right)}}{48EI} \\ \end{aligned}$$

20 $$\begin{aligned} \theta \left( x \right) & = {\Gamma }_{zx} {\upeta }\frac{{37l^{4} - 90l^{2} L^{2} + 60l^{3} x - 180lL^{2} x - 15x^{2} \left( {6L^{2} - x^{2} } \right)}}{360EI} \\ & \,\,\,\,\, + g_{z} \left( 0 \right){\upeta }\frac{{3l^{3} - 12l^{2} L + 18lL^{2} + 8x\left( {3L^{2} + 3Lx + x^{2} } \right)}}{48EI} \\ \end{aligned}$$

$$- l \le x \le 0$$

21 $$Z\left( {\text{x}} \right) = {\Gamma }_{zx} {\upeta }\frac{{x\left( {7l^{4} - 10l^{2} x^{2} + 3x^{4} } \right)}}{360EI} - g_{z} \left( 0 \right){\upeta }\frac{{\left( {l + x} \right)x^{2} \left( {3l^{2} + 6L^{2} - 2l\left( {6L + x} \right)} \right)}}{48EIl}$$

22 $$\theta \left( x \right) = {\Gamma }_{zx} {\upeta }\frac{{7l^{4} - 30l^{2} x^{2} + 15x^{4} }}{360EI} - g_{z} \left( 0 \right){\upeta }\frac{{x\left( {6l^{3} + 18L^{2} x - 3l^{2} \left( {8L - x} \right) + 4l\left( {3L^{2} - 9Lx - 2x^{2} } \right)} \right)}}{48EIl}$$

One can find the condition upon which the beam’s displacements at its free ends under the uniform force of gravity vanish ($L l$). One has

23 $$Z\left( L \right) = g_{z} \left( 0 \right){\upeta }\frac{{\left( {l - L} \right)\left( {l^{3} - 6l^{2} L + 12lL^{2} - 6L^{3} } \right)}}{48EI} = 0$$

It yields

24 $$l = \left( {2 - 2^{1/3} } \right)L$$

**Figure 2:**
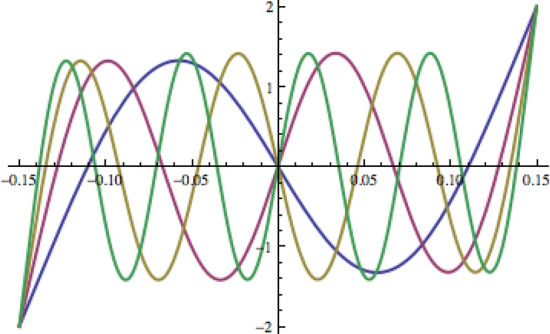
Mirror-symmetric eigenmodes of a free-free 30 cm thin beam. The first, second, third and fourth mirror-symmetric eigenmodes are depicted in blue, maroon, gold and green consequently. The eigenmode’s zero-crossing locations are at 0, ± 11.0368 cm compared to 0, ± 11.1012 cm derived from Eq. (24) for the free-hinged-hinged-hinged-free beam of the same length.

It is interestingly enough to note that the positions ± $l$ in Eq. (24) match closely with free-free beam’s nodal locations of the first mirror-symmetric eigenmode as shown in Fig. 2 below^19^.

The deflections of the free-hinged-hinged-hinged-free beam’s ends with the locations of the hinges as per Eq. (24) are as follows

25 $$Z\left( L \right) = - Z\left( { - L} \right) = - 2.2 \times 10^{ - 3} {\Gamma }_{zx} {\upeta }\frac{{L^{5} }}{EI} \cong - 10^{ - 3} {\Gamma }_{zx} \frac{{mL^{4} }}{EI}$$

where *m* is the total mass of the beam and *mL*^4^*/EI* can be treated as the beam’s intrinsic gradiometric gain having the dimension of the product of time squared and distance. It is also interesting to note that this parameter can be measured quite accurately for particular dimensions and material. As an example, the product *EI* can be extracted from measuring the resonant frequencies (say, the first resonant mode) of a standard cantilever fixed-free beam of the same material and cross section. In turn, this means that absolute gravity gradient measurements are possible provided that the deflection of the beam from its force-free position can also be calibrated in absolute units by measuring them at the locations (calibration stations) where the local gravity gradients are well known.

**Figure 3:**
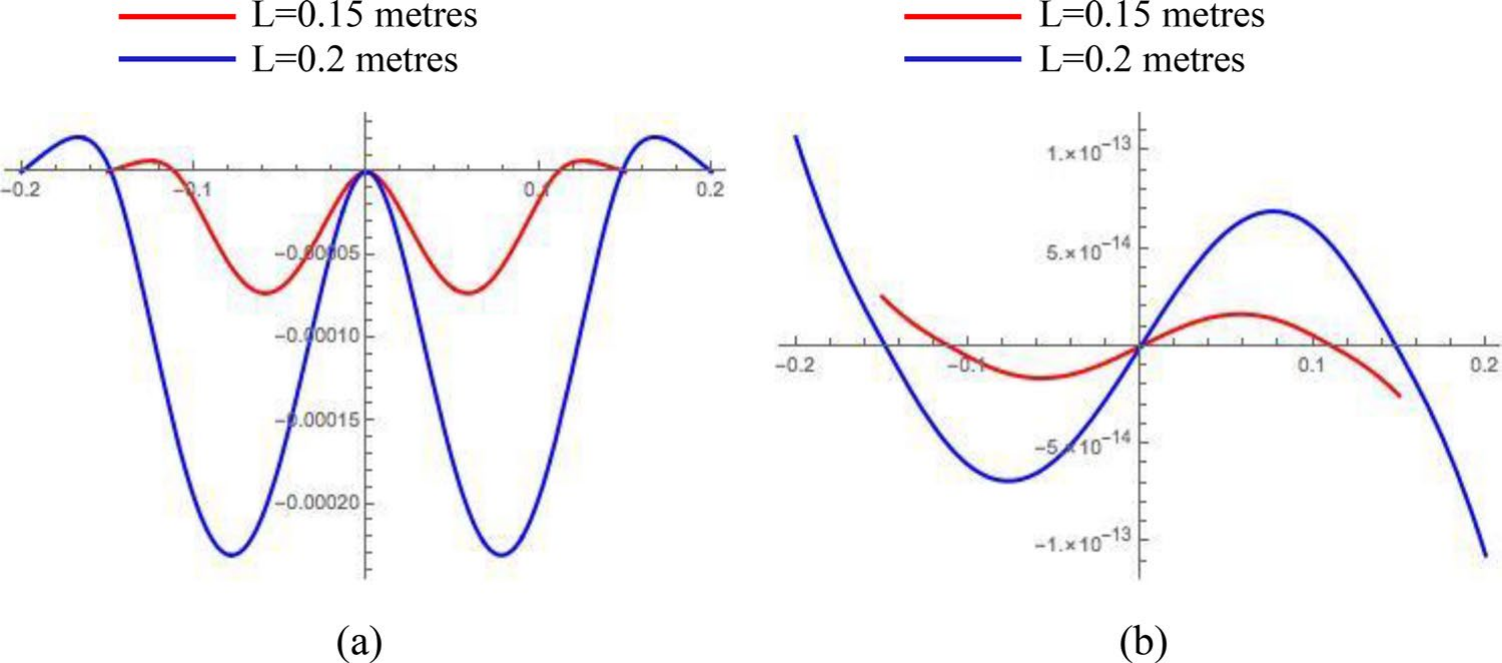
(**a**) – the symmetric deflection of the free-hinged-hinged-hinged-free beam under the full projection of the Earth’s gravity; (**b**) – the mirror-symmetric deflection of the beam under 10E gravity gradient (all numbers are in metres, material is Phosphor Bronze).

In Fig. 3 below, two beam’s spatial profiles show its deflection under full-g body load and, independently, under 10E gravity gradient along its length (g = − 9.8 m/s^2^ is the Earth’s gravitational acceleration chosen to be directed downwards, 1E = 1Eotvos = 10^–9^ 1/s^2^ is the unit of gravity gradients, *b* = *0.01 *m,   *d= 0.00025 * m, $l = \pm \left( {2 - 2^{1/3} } \right)L$).

## Design and manufacturing error analysis (static)

Manufacturing processes are not perfect, and the ideal scenario outlined above can be modified to account for several imperfections, including:

- Asymmetric positioning of the left–right side hinges with respect to the central one, so the beam’s spans are not equal to each other;
- The beam’s mass per unit length is not the same at every cross-section of the beam (major contributions are beam’s manufacturing tolerances and its density variations);

One can prove that all such effects result in mixing the true gravity gradient related deflection of the beam with the one caused by the uniform force of gravity distribution. As an example, the deflection of the beam’s right end, including an error term caused by the mispositioning of the right-side hinge $\ell + \delta \ell$ and asymmetric right end $L + \delta L$, is as follows

26 $$Z\left( L \right) \cong - 10^{ - 3} {\Gamma }_{zx} \frac{{mL^{4} }}{EI} + g_{z} \left( 0 \right) \frac{{mL^{3} }}{96EI}\left( {\alpha \frac{\delta \ell }{L} + \beta \frac{\delta L}{L}} \right) + Z_{0} \left( L \right)$$

where $\alpha$ and $\beta$ are numerical factors of the order of unity and Z_0_ is a zero-force offset due to a residual stress embedded into the beam. In real life, there always will be some small residual curvature of the beam’s plane due to the remaining (built-in) stress embedded into its atomic structure and caused by its history of mechanical and thermal treatment during manufacturing. If the stress is constant (frozen) in time, then this appears as the presence of a constant uniform load or a constant gravity gradient along the ribbon’s length independent of the environmental conditions. These will result in a systematic error in measuring gravity gradients in absolute units, namely, a measurement bias—a difficult situation. As with most sensitive equipment, relative measurements are much easier to deal with provided the relevant gradiometer’s set-up is stable—the requirement that makes the development of practical gravity gradiometers look similar to ”impossible” grade missions. However, in the case of such spatially distributed flexible sensing element, i.e. a free-hinged-hinged-hinged-free beam, the biased deflections of its different spans can be measured independently and combined (in real time or in a post-processing stage) in such a way that this would cancel out the systematic errors mentioned above. A simple way of measuring and maximally removing the systematic errors is to rotate the sensor in the horizontal plane by 180 degree and sum-up the results of the measurements. The $g_{z}$ proportional term will not change its sign while the gradient term will be eliminated due to its asymmetry along the X-axis. Combining dynamic deflections of the sensor’s different spans would also cancel out such dynamic effects (see the dynamic analysis section below) as mixing desired signals with linear acceleration if a gradiometer is mounted on a moving platform (airborne gravity gradiometry as an example). The latter is possible since the forced mechanical displacements (either resonant or non-resonant) of every infinitesimally small cross section of vibrating beams are superposed as a weighted sum of all possible eigenmodes, formed by beam’s boundary conditions, and representing true standing waves^20^.

**Figure 4:**
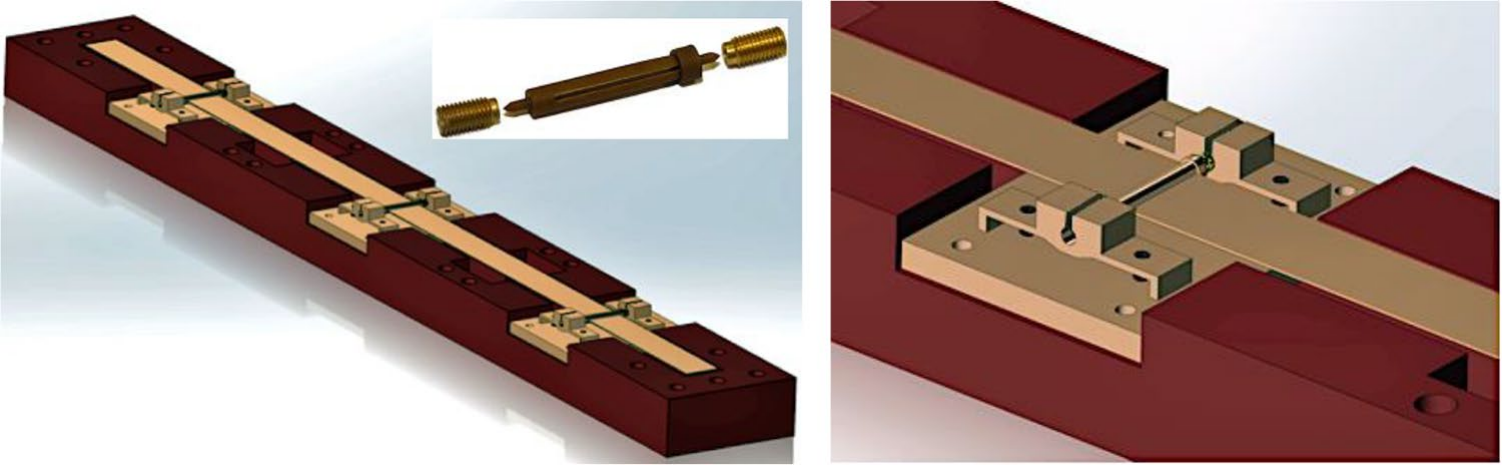
A free-hinged-hinged-hinged-free beam made by sliding a straight long ribbon into ultra-small and almost weightless shafts manufactured by experienced wrist-watch makers [http://www.wcawa.org.au]. The shafts are locked from both sides and held inside sapphire-bearing spring-loaded threaded insertions fixed in shaft holders at nodal positions along the beam’s length within a few micrometres tolerance. The thermal expansion coefficients of all materials used in this assembly including fasteners are closely matched.

The design and manufacture of free-hinged-hinged-hinged-free beams is a challenging engineering problem. The analysis above assumes the hinges are solid structures that do not possess any intrinsic torsional spring constant. Such hinges can be made as solid micro-shafts allowing only free rotation of the beam around the nodal axes and connected to an external frame of reference in a manner that used in the precision wrist-watch making technique, e.g. certified mechanical chronometers. Jewel-bearing spring-loaded insertions using synthetic sapphire and holding rotating shafts have been widely used in precision instruments where extremely low friction, long life and dimensional accuracy are important. The latter also allows for a self-alignment of the beam’s nodal axes along the zero-force plane (XOY in the case above). Such technique can also mitigate a residual mismatch of the thermal expansion, caused by thermal gradients, of the materials used to make a beam, shafts and their holders and the frame of reference. All such materials must have very closely matched thermal expansion coefficients. A typical way of further reducing the effects of thermal gradients around the sensor assembly is to keep it in a medium level vacuum or in a locked space filled with gaseous helium at a low pressure. The latter provides almost instant thermal alignment along the sensor assembly due to its ultra-fast speed of establishing thermal equilibrium at optimum pressure values^32^. An example of a solid shaft-based design of a free-hinged-hinged-hinged-free beam is depicted in Fig. 4 below.

**Figure 5:**
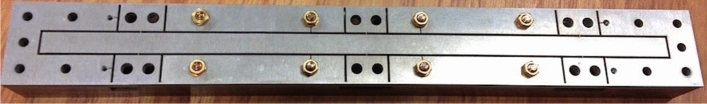
A free-hinged-hinged-hinged-free beam made by precision EDM process. The whole structure is wire cut with about 3–5 microns tolerance from a single piece of flat metal foil. In this case, the hinges represent micro-pivots of an optimum cross section connecting the middle flexible section (ribbon) to the rest of the frame. Material shown is a composite Tungsten/Copper alloy.

Another design of a free-hinged-hinged-hinged-free beam is depicted in Fig. 5 below. The whole structure is EDM wire cut with about 3–5 microns tolerance from a single piece of flat metal foil. In this case, the hinges represent micro-pivots of an optimum cross section connecting the middle flexible section (ribbon) to the rest of the foil that form a zero-force frame of reference. In turn, the latter can be firmly mounted upon another solid frame made of the same material as the foil. The pivots possess an intrinsic torsional spring constant and this changes the boundary conditions of the true hinged beam design^21,22^. Analytical evaluation becomes a bit more complicated as the additional torsional rigidity is not well known and must therefore be idealized. During the EDM processing time, the fine pivot structure experiences not well-defined treatment by heating the foil material in a water basin, which is the standard processing environment for the EDM technique. The effect of the latter upon a particular chosen material is not well known either. The cutting wire material does matter as well. Software modelling is needed to simulate these effects and compare the results with experimental data. A detailed error analysis of a prototype gravity gradiometer along with experimental data will be published elsewhere.

## Dynamic analysis of free-hinged-hinged-hinged-free beam

The dynamic analysis of free-hinged-hinged-hinged-free beam is different for the Rayleigh-Timoshenko theory and for the Euler–Bernoulli-Lagrange theory. Timoshenko introduced corrections to the latter taking into account rotary inertia and shear deformation which leads to two linear differential equations for beam’s transverse deflection and its bending slope^23^. This gives better results in dynamic analyses for beam’s high frequency eigenmodes. However, for the multi-span thin beam under consideration the latter are vastly suppressed and only a few low frequency eigenmodes do matter for practical applications. Assuming the Euler–Bernoulli-Lagrange theory is still accurate enough to analyse the dynamic behaviour of the beam under consideration^24^, one has the following dynamic Euler–Lagrange equations for the beam’s transverse displacement and bending slope

27 $$\eta \frac{{\partial^{2} Z}}{{\partial t^{2} }} + h\frac{\partial Z}{{\partial t}} + EI\frac{{\partial^{4} Z}}{{\partial x^{4} }} = - \eta a\left( t \right) + \eta \tilde{\Gamma }_{zx} x + F_{L} \left( {x,t} \right)$$

28 $$\eta \frac{{\partial^{2} \theta }}{{\partial t^{2} }} + h\frac{\partial \theta }{{\partial t}} + EI\frac{{\partial^{4} \theta }}{{\partial x^{4} }} = \eta \tilde{\Gamma }_{zx} + \frac{\partial }{\partial x}F_{L} \left( {x,t} \right)$$

29 $$\tilde{\Gamma }_{zx} = - \frac{{d\Omega_{y} }}{dt} - \Omega_{z} \Omega_{x}$$

where *h* is the coefficient of friction per unit length (assumed to be the same along the beam’s length), *F*_*L*_*(x,t)* is the effective Langevin force *per unit length* forcing the beam to stay in thermal equilibrium with external environment with temperature *T*, *a(t)* and $\Omega_{X,Y,Z}$ are kinematic (linear) acceleration normal to the beam’s surface and angular velocity vector components of the beam’s reference frame respectively. The term $\tilde{\Gamma }_{zx}$ in the right side of Eqs. (27) and (28) is called a dynamic gradient that affects the measurement of real gravity gradients by gravity gradiometers mounted on moving platforms representing non-inertial reference frames^25^.

As the beam represents a multi-mode mechanical resonator, its response to the noise driving force is not the same as that of a Brownian particle. The intensity of the effective Langevin force per unit length should be calculated from the condition that the mean energy for each mode *n* of the resonator will be given by $\left\langle {W_{n} } \right\rangle = k_{B} T$ ^26^. A wave-function analysis applied to the free-hinged-hinged-hinged-free beam is presented below (see also^27,28^). One has

30 $$Z\left( {x,t} \right) = \mathop \sum \limits_{n} \alpha_{n} \left( t \right) \psi_{n}^{\left( + \right)} \left( x \right) + \mathop \sum \limits_{n} \beta_{n} \left( t \right)\psi_{n}^{\left( - \right)} \left( x \right)$$

31 $$\mathop \int \limits_{ - L}^{L} dx\psi_{n}^{\left( + \right)} \left( x \right)\psi_{m}^{\left( + \right)} \left( x \right) = 2L\delta_{mn} , \mathop \int \limits_{ - L}^{L} dx\psi_{n}^{\left( - \right)} \left( x \right)\psi_{m}^{\left( - \right)} \left( x \right) = 2L\delta_{mn}$$

32 $$\mathop \int \limits_{ - L}^{L} dx\psi_{n}^{\left( + \right)} \left( x \right)\psi_{m}^{\left( - \right)} \left( x \right) = 0, \delta_{mn} = 0 m \ne n, \delta_{nn} = 1$$

The modal wave-functions $\psi_{n}^{\left( + \right)} \left( x \right)$ and $\psi_{n}^{\left( - \right)} \left( x \right)$ represent symmetric and mirror-symmetric eigenfunctions satisfying the following relations

33 $$\psi_{n}^{\left( + \right)} \left( x \right) = \psi_{n}^{\left( + \right)} \left( { - x} \right), \psi_{n}^{\left( - \right)} \left( x \right) = - \psi_{n}^{\left( - \right)} \left( { - x} \right)$$

These eigenfunctions are found by solving the following characteristic equation for each of the beam’s span

34 $$\frac{{d^{4} }}{{dx^{4} }}\psi_{n}^{\left( \pm \right)} \left( x \right) = k_{\left( \pm \right),n}^{4} \psi_{n}^{\left( \pm \right)} \left( x \right)$$

where $k_{\left( \pm \right),n}$ are the partial modal eigenvalues that depend on specific sets of boundary and continuity conditions corresponding to the beam’s force-free vibration modes. The orthogonality of the modal eigenfunctions is automatically provided by the beam’s free ends boundary conditions (Eq. (35) and  (36) below) and by their symmetry (Eq. (33))

35 $$\frac{{d^{2} \psi_{n}^{\left( \pm \right)} }}{{dx^{2} }}|_{x = - L} = \frac{{d^{2} \psi_{n}^{\left( \pm \right)} }}{{dx^{2} }}|_{x = L} = 0$$

36 $$\frac{{d^{3} \psi_{n}^{\left( \pm \right)} }}{{dx^{3} }}|_{x = - L} = \frac{{d^{3} \psi_{n}^{\left( \pm \right)} }}{{dx^{3} }}|_{x = L} = 0$$

The boundary and continuity conditions at $\pm \ell$ nodal locations for either symmetric or mirror-symmetric eigenfunctions are as follows

37 $$\psi_{n}^{\left( \pm \right)} \left( { - l} \right) = \psi_{n}^{\left( \pm \right)} \left( l \right) = 0$$

38 $$\frac{{d\psi_{n}^{\left( \pm \right)} }}{dx}|_{l + \varepsilon } = \frac{{d\psi_{n}^{\left( \pm \right)} }}{dx}|_{l - \varepsilon } , \varepsilon \to 0$$

39 $$\frac{{d\psi_{n}^{\left( \pm \right)} }}{dx}|_{ - l - \varepsilon } = \frac{{d\psi_{n}^{\left( \pm \right)} }}{dx}|_{ - l + \varepsilon } , \varepsilon \to 0$$

40 $$\frac{{d^{2} \psi_{n}^{\left( \pm \right)} }}{{dx^{2} }}|_{l - \varepsilon } = \frac{{d^{2} \psi_{n}^{\left( \pm \right)} }}{{dx^{2} }}|_{l + \varepsilon } = 0, \varepsilon \to 0$$

41 $$\frac{{d^{2} \psi_{n}^{\left( \pm \right)} }}{{dx^{2} }}|_{ - l - \varepsilon } = \frac{{d^{2} \psi_{n}^{\left( \pm \right)} }}{{dx^{2} }}|_{ - l + \varepsilon } = 0, \varepsilon \to 0$$

For the central hinged location (*x* = 0) one has

42 $$\psi_{n}^{\left( \pm \right)} \left( 0 \right) = \psi_{n}^{\left( \pm \right)} \left( 0 \right) = 0$$

43 $$\frac{{d\psi_{n}^{\left( + \right)} }}{dx}|_{0 - \varepsilon} = - \frac{{d\psi_{n}^{\left( + \right)} }}{dx}|_{0 + \varepsilon } , \varepsilon \to 0$$

44 $$\frac{{d^{2} \psi_{n}^{\left( + \right)} }}{{dx^{2} }}|_{0 - \varepsilon} = \frac{{d^{2} \psi_{n}^{\left( + \right)} }}{{dx^{2} }}|_{0 + \varepsilon } = 0, \varepsilon \to 0$$

45 $$\frac{{d\psi_{n}^{\left( - \right)} }}{dx}|_{0 - \varepsilon} = \frac{{d\psi_{n}^{\left( - \right)} }}{dx}|_{0 + \varepsilon } , \varepsilon \to 0$$

46 $$\frac{{d^{2} \psi_{n}^{\left( - \right)} }}{{dx^{2} }}|_{0 - \varepsilon} = \frac{{d^{2} \psi_{n}^{\left( - \right)} }}{{dx^{2} }}|_{0 + \varepsilon } = 0, \varepsilon \to 0$$

After substituting Eq. (29) into Eq. (27) and performing trivial mathematical calculations, one finds

47 $$\frac{{d^{2} \alpha_{n} }}{{dt^{2} }} + \frac{2}{\tau }\frac{{d\alpha_{n} }}{dt} + \omega_{\left( + \right),n}^{2} \alpha_{n} = - a\left( t \right)\frac{1}{2L}\mathop \int \limits_{ - L}^{L} dx\psi_{n}^{\left( + \right)} + \frac{1}{m}\mathop \int \limits_{ - L}^{L} dx\psi_{n}^{\left( + \right)} F_{L} \left( {x,t} \right)$$

48 $$\frac{{d^{2} \beta_{n} }}{{dt^{2} }} + \frac{2}{\tau }\frac{{d\beta_{n} }}{dt} + \omega_{\left( - \right),n}^{2} \beta_{n} = \frac{1}{2L}\tilde{\Gamma }_{zx} \mathop \int \limits_{ - L}^{L} dxx\psi_{n}^{\left( - \right)} + \frac{1}{m}\mathop \int \limits_{ - L}^{L} dx\psi_{n}^{\left( - \right)} F_{L} \left( {x,t} \right)$$

where $\tau = 2\eta /h$ is the beam’s relaxation time and $\omega_{\left( \pm \right),n} = 2\pi f_{\left( \pm \right),n}$ are its free-vibration angular resonant frequencies^29^

49 $$f_{\left( \pm \right),n} = \frac{1}{2\pi }\sqrt {\frac{EI}{\eta }} k_{\left( \pm \right),n}^{2}$$

The eigenvalues $k_{\left( \pm \right),n}$ are unambiguously determined from Eq. (34) which is manifestly invariant of the choice of normalisation of modal eigenfunctions

50 $$\psi_{n}^{\left( \pm \right)} \left( x \right) \to N\psi_{n}^{\left( \pm \right)} \left( x \right)$$

**Figure 6:**
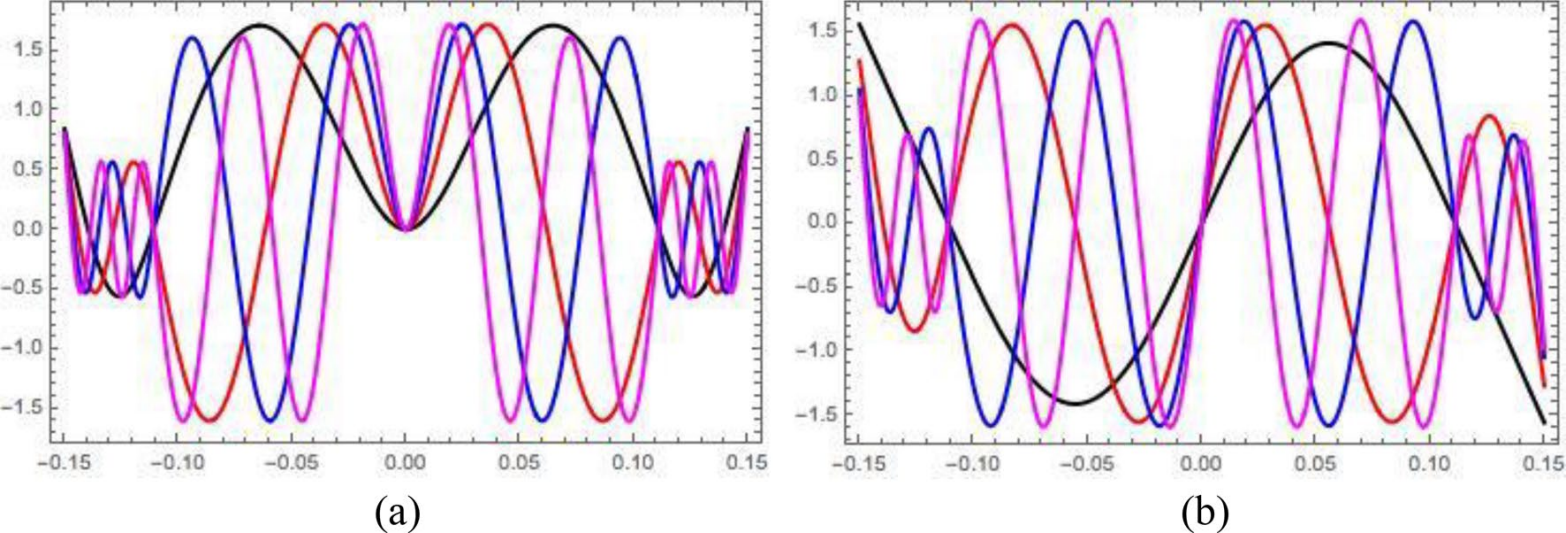
(**a**) Symmetric eigenmodes of free-hinged-hinged-hinged-free beam (1st—black, 2nd—red, 3rd—blue, 4th—magenta); (**b**) Mirror-symmetric eigenmodes of free-hinged-hinged-hinged-free beam (1st—black, 2nd—red, 3rd—blue, 4th—magenta).

The exact solutions of the Eq. (33) for the free-hinged-hinged-hinged-free beam are presented in Supplementary Materials section of this article. The first four force-free eigenmodes of the beam under consideration are depicted in Fig. 6 below for *L* = *0.15* m and the locations of side hinges $l = \pm \left( {2 - 2^{1/3} } \right)L$:

## Noise only driven free-hinged-hinged-hinged-free beam

The total energy of the free-hinged-hinged-hinged-free beam under consideration consists of a kinetic term and the potential energy stored in the beam’s atomic structure^29^

51 $$W = \frac{\eta }{2}\mathop \int \limits_{ - L}^{L} dx\left( {\frac{\partial Z}{{\partial t}}} \right)^{2} + \frac{EI}{2}\mathop \int \limits_{ - L}^{L} dx\left( {\frac{{\partial^{2} Z}}{{\partial x^{2} }}} \right)^{2}$$

By integrating the potential energy term in Eq. (51) by parts and replacing the beam’s transverse displacement *Z(x,t)* by its modal series expansion in Eq. (29) (the beam’s free-ends boundary conditions are taken into account), one finds

52 $$W = \mathop \sum \limits_{n} \left( {W_{\left( + \right),n} + W_{\left( - \right),n} } \right) = \frac{m}{2}\mathop \sum \limits_{n} \left[ {\left( {\frac{{d\alpha_{n} }}{dt}} \right)^{2} + \left( {\frac{{d\beta_{n} }}{dt}} \right)^{2} + \omega_{\left( + \right),n}^{2} \alpha_{n}^{2} + \omega_{\left( - \right),n}^{2} \beta_{n}^{2} } \right]$$

Equation (52) above is the standard representation of the total energy of multi-mode mechanical oscillator where $\alpha_{n}$ and $\beta_{n}$ are its modal mechanical displacement amplitudes. The spectral densities of the latter for the noise only driven beam under consideration are as follows

53 $$S_{\left( \pm \right),n} \left( \omega \right) = \frac{{D_{F} }}{{\left( {\omega^{2} - \omega_{\left( \pm \right),n}^{2} } \right)^{2} + \left( {\frac{2\omega }{\tau }} \right)^{2} }}\frac{2L}{{m^{2} }}$$

where *D*_*F*_ is the intensity of the Langevin force:

54 $$F_{L} \left( {x,t} \right)F_{L} \left( {x^{^{\prime}} ,t^{^{\prime}} } \right) = D_{F} \delta \left( {x - x^{^{\prime}} } \right)\delta \left( {t - t^{^{\prime}} } \right)$$

If the beam is in thermal equilibrium with its environment with temperature *T*, the following requirement holds^30^

55 $$W_{\left( \pm \right),n} = \frac{{D_{F} }}{2\pi \eta }\mathop \int \limits_{0}^{\infty } d\omega \frac{{\omega^{2} + \omega_{\left( \pm \right),n}^{2} }}{{\left( {\omega^{2} - \omega_{\left( \pm \right),n}^{2} } \right)^{2} + \left( {\frac{2\omega }{\tau }} \right)^{2} }} = k_{B} T$$

The integration in Eq. (55) yields

56 $$D_{F} = 2k_{B} Th$$

It is possible now to estimate the thermal fluctuations imposed by the Langevin force upon, say, the mechanical displacement of the beam’s free ends under a gravity gradient along its length. In turn, this would allow for an estimate of the thermal limit in measuring gravity gradients by the primary sensing element such as free-hinged-hinged-hinged-free beam. One finds from Eqs. (30), (53) and (56)

57 $$S\left( {L,\omega } \right) = \mathop \sum \limits_{n} S_{\left( + \right),n} \left( \omega \right)\psi_{n}^{\left( + \right)2} \left( L \right) + \mathop \sum \limits_{n} S_{\left( - \right),n} \left( \omega \right)\psi_{n}^{\left( - \right)2} \left( L \right)$$

where $S\left( {L,\omega } \right)$ is the displacement spectral noise of the beam’s free ends. Considering the quasi-static approximation only ($\omega \to 0$), one has

58 $$S^{1/2} \left( {L,\omega } \right) = \sqrt {\frac{{4k_{B} T}}{m\tau }\left( {\mathop \sum \limits_{n} \frac{{\psi_{n}^{\left( + \right)2} \left( L \right)}}{{\omega_{\left( + \right),n}^{4} }} + \mathop \sum \limits_{n} \frac{{\psi_{n}^{\left( - \right)2} \left( L \right)}}{{\omega_{\left( - \right),n}^{4} }}} \right)} \frac{metres}{{\sqrt {Hz} }}$$

By combining Eqs. (58), (49) and (25), one finds the equivalent gravity gradient spectral noise

59 $$S_{{\Gamma }}^{1/2} \left( {L,\omega } \right) = \frac{{10^{12} }}{{L^{5} }} \sqrt {\frac{{k_{B} T}}{m\tau }\left( {\mathop \sum \limits_{n} \frac{{\psi_{n}^{\left( + \right)2} \left( L \right)}}{{k_{\left( + \right),n}^{8} }} + \mathop \sum \limits_{n} \frac{{\psi_{n}^{\left( - \right)2} \left( L \right)}}{{k_{\left( - \right),n}^{8} }}} \right)} \frac{Eotvos}{{\sqrt {Hz} }}$$

It is worth noting that either Eqs. (58) or (59) are invariant of the eigenfunction normalisation factor in Eq. (50) (chosen to be $\sqrt {2L}$) as in the dynamic analysis the total mass of the beam is defined as

60 $$m = \eta \mathop \int \limits_{ - L}^{L} dx\psi_{n}^{\left( \pm \right)} \left( x \right)\psi_{n}^{\left( \pm \right)} \left( x \right) = 2L\eta$$

For the over-constrained free-hinged-hinged-hinged-free beam under consideration only the first (*n* = 1) mirror-symmetric eigenmode $\psi_{n}^{\left( - \right)}$ gives the major contribution to the right sides of Eqs. (59) and  (60). In case differential displacement measurements are used to measure relative motion of the beam’s free ends (*Z*(*L*) − *Z*( − *L*)), then 3 dB noise reduction should be applied to Eq. (59). Table 1 below gives a few examples for a particular beam’s dimensions, materials, and the locations of the side hinges at $l = \pm \left( {2 - 2^{1/3} } \right)L$.

## Paired static accelerometers versus free-hinged-hinged-hinged-free beam

The Common Mode Rejection Ratio (CMRR) in its classic meaning applies to any four-port measuring device where two independent non-measurable physical signals are applied to two input ports and then converted via input transfer functions into two output signals representing measurable physical quantities such as electric voltage. Non-measurable input signals can be atom-size mechanical displacements, gravitational and linear accelerations, temperature, pressure, light and the like. As an example, a relative gravity gradiometer can be set up as a pair of linear static accelerometers, separated by a base line, where their output signals are subtracted in real time or in a post processing stage (output voltages can be digitized and analytically differentiated).

It must be noted that any single static accelerometer has zero CMRR. If paired, the differential output signal is as follows

61 $$\Delta V = V_{1} - V_{2} = k_{1} g_{1} - k_{2} g_{2} = \overline{k}\left( {g_{1} - g_{2} } \right) + \overline{g}\left( {k_{1} - k_{2} } \right)$$

where *k*_*1*_ and *k*_*2*_ are the accelerometer transfer functions, *g*_*1*_ and *g*_*2*_ are the gravitational accelerations at the two different locations occupied by the accelerometers separated by some base line, and

62 $$\overline{k} = \frac{{k_{1} + k_{2} }}{2}, \overline{g} = \frac{{g_{1} + g_{2} }}{2}$$

The CMRR in this case is

63 $$CMRR \cong 6 + 20Log\left( {\frac{{k_{1} - k_{2} }}{{k_{1} + k_{2} }}} \right) dB$$

In gravity gradiometer design pursuits based on paired static accelerometers, transfer functions are matched electronically with a high precision and included in feedback loops to keep the balance stable^33^. Without any electronic enhancement, the transfer functions (gains) of precision accelerometers can be matched to within a few parts in ten thousand at best^34^. This results in the “initial” CMRR of approximately  − 70 dB or less.

In the case of free-hinged-hinged-hinged-free beam, Eq. (26) combined with Eq. (4) can be represented in a form like Eq. (61)

64 $$Z\left( L \right) = \overline{k}_{int} \left( {g_{z} \left( L \right) - g_{z} \left( { - L} \right)} \right) + \overline{g}\left( {k_{int} \left( L \right) - k_{int} \left( { - L} \right)} \right) + Z_{0} \left( L \right)$$

where

65 $$\overline{k}_{int} \cong 5 \left( {10} \right)^{ - 4} \frac{{mL^{3} }}{EI}$$

66 $$\overline{g} = \frac{{g_{z} \left( L \right) + g_{z} \left( { - L} \right)}}{2}$$

and

67 $$k_{int} \left( L \right) - k_{int} \left( { - L} \right) \cong 5 \left( {10} \right)^{ - 3} \frac{{mL^{3} }}{EI}\left( {\alpha \frac{\delta \ell }{L} + \beta \frac{\delta L}{L} + \gamma \frac{\delta m}{m}} \right)$$

It is worth noting again that the mechanical displacement function *Z*(*L*) in Eqs. (26) and (64) is a non-measurable quantity as no mechanical displacement sensing means are applied to free-hinged-hinged-hinged-free beam as the primary sensing element. The corresponding "internal" CMRR is

68 $$CMRR \cong 20 + 20Log\left( {\alpha \frac{\delta \ell }{L} + \beta \frac{\delta L}{L} + \gamma \frac{\delta m}{m}} \right) dB , \alpha ,\beta ,\gamma \approx 1$$

In Eq. (68) a new term *δm*/*m* is added, which represents a beam’s symmetry breaking point mass deposited at the positive end of the free-hinged-hinged-hinged-free beam. A non-uniform beam’s width and/or non-uniform mass per-unit-length can be reliably modelled by this local mass deposition^35^. One can argue that vertical misalignment of a nodal position, say the central one, will not contribute to the CMRR above as the beam’s symmetry is not broken for that case. There is always a plane defined through any two nodal axes so only one vertical misalignment needs to be considered. For typical machining tolerances, the latter is small enough such that it only increases the stiffness of a free-hinged-hinged-hinged-free beam’s vibration modes as a second order effect.

It is interesting to note that a negative point mass can be physically added to either side of the beam by using precision laser ablation (trimming)^36^. This can counterbalance the small errors caused by the misalignment of the nodal axes along the beam’s length and greatly increase the CMRR. It is feasible to achieve at least − 60 dB CMRR at the manufacturing stage and then another − 120 dB CMRR by using precision electronic gain matching techniques and other well-known means to get up to − 180 dB CMRR needed for mobile gravity gradiometry applications^6^.

## Concluding remarks

The static and dynamic analysis of the free-hinged-hinged-hinged-free beam is the first step in presenting a novel design of a primary sensing element having a single sensitivity axis that can be used in future advanced gravity gradiometers. The latter should be capable of operating in any orientation with respect to the Earth’s acceleration of gravity and in a limited space that is typical for such unmanned mobile platforms as UAVs (either airborne or submersible) and drones. From Table 1 above it follows that the beam’s length is the most critical factor for either increasing its sensitivity to a gravity gradient along its length or reducing the thermally activated gravity gradient noise. The latter is the limiting factor for this type of gravity gradiometer. A median baseline (2L) of 0.35 m, based on the free-hinged-hinged-hinged-free beam and made of a Tungsten/Copper composite alloy, would provide better than 10 Eotvos resolution at 3 s measurement time. The corresponding mechanical displacement measurements at the level of ~ 10^–14^ m/√Hz can be provided by a number of currently available room-temperature techniques (capacitive, microwave, optical) including new emerging techniques such as quantum and quantum enabled sensing^31^. As all existing gravity and gravity gradient front-end sensing elements, including the proposed triple-hinged beam design, can only provide quasi-static mechanical displacement measurements, the measured data sets always contain 1/f noise (zero-point drift) caused by temperature instabilities, mechanical creep in the primary sensing element, voltage drift in operational amplifiers and other electronic components. These effects can be removed by applying a modulation-demodulation technique^6,7^ that can shift the operational frequency bandwidth to a white noise area where maximum signal-to-noise ratio is provided (this approach is currently under development and will be discussed elsewhere). The most important feature of the distributed sensing element as the free-hinged-hinged-hinged-free beam is that it allows for simultaneous measurement of the dynamic displacements of the beam’s different spatial locations representing true standing waves. If combined in a proper manner, this could cancel out the effect of one of the most disturbing factors for gravity gradiometers, if being deployed on moving platforms, namely large kinematic and uniform gravitational accelerations applied to the primary sensing element and its frame of reference. This approach is entirely new in gravity gradiometry and may open the door to the most desired use of gravity gradiometers in the strapped-down mode onboard commercial drones and other unmanned platforms.

**Table 1:**
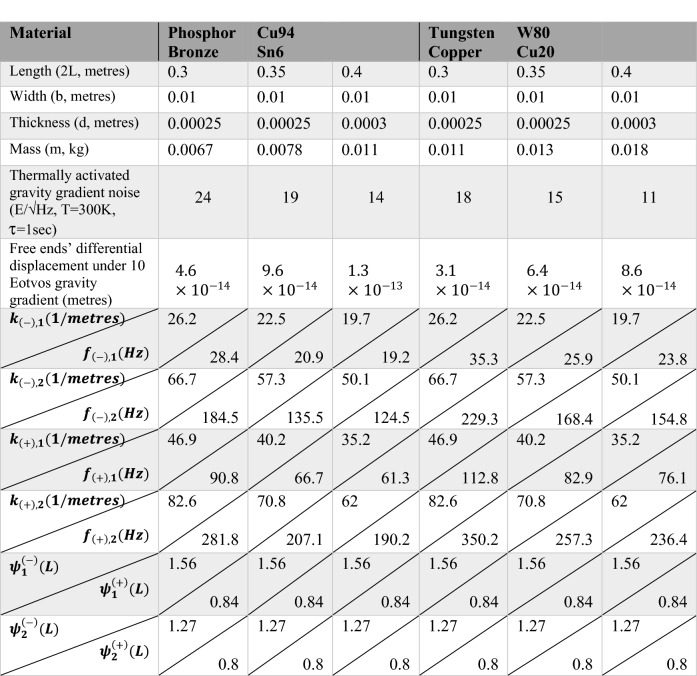
Numerical parameters of free-hinged-hinged-hinged-beam with different lengths and cross sections for two different materials (Phosphor Bronze and Tungsten Copper alloys); Calculated the first two modal eigenvalues and mechanical resonant frequencies for both mirror-symmetric and symmetric eigenfunctions; calculated differential mechanical displacement of the beam’s free ends under 10 Eotvos gravity gradient and the thermally activated equivalent gravity gradient noise.

## Supplementary Information

Supplementary Information.

## References

[1] Servarajan, A. *et al.* Analysis of a nonuniform cantilever beam MOEM accelerometer under closed loop operation. Proc. SPIE 5763, Smart Structures and Materials: Smart Electronics, MEMS, BioMEMS, and Nanotechnology (2005).

[2] Jiang, X. *et al*. An integrated surface micromachined capacitive lateral accelerometer with 2μG/√Hz resolution. Solid-state sensor, actuator and microsystems workshop (Hilton Head Island, South Carolina, 2–6 June 2002) 202–205 (2002).

[3] Liu H, Pike WT, Dou G (2016). A seesaw-lever force-balancing suspension design for space and terrestrial gravity gradient sensing. J. Appl. Phys..

[4] Wang C (2020). Micromachined accelerometers with sub-μg/Hz noise floor: a review. Sensors.

[5] Golden H, McRay W, Veryaskin AV (2007). Description of and Results from a Novel Borehole Gravity Gradiometer.

[6] Veryaskin, A.V. Gravity, magnetic and electromagnetic gradiometry: strategic technologies in the 21st century. Institute of physics publishing (IoP, UK), Second Edition, 183 (2021).

[7] Veryaskin, A.V. *et al*. Intrinsic gravity gradiometer and gravity gradiometry, PCT Patent WO2018071993A1 (2017), US Patent (granted) 11,002,878 (2021).

[8] Lamb, H. Statics. 325 (Cambridge University Press, 1960).

[9] Lorenzini EC, Gullahorn GE, Fuligni F (1988). Recent developments in gravity gradiometry from the space-shuttle-borne tethered satellite system. J. Appl. Phys..

[10] Tian W (2012). High resolution space quartz-flexure accelerometer based on capacitive sensing and electrostatic control technology Rev. Sci. Instrum..

[11] Timoshenko, S., Young, D. Vibration problems in engineering, (Wiley, New York, 1974).

[12] Rayleigh, J.W.S. Baron: The Theory of Sound, Vol. 2, (Macmillan Press, 1896)

[13] Timoshenko SP (1921). On the correction factor for shear of the differential equation for transverse vibrations of prismatic bar. Philos. Mag..

[14] Rosinger HE, Ritchie GI (1977). On Timoshenko's correction for shear in vibrating isotropic beams. J. Phys. D Appl. Phys..

[15] Schaefer, C. Einfuhrung in die theoretische physik zweiter band Berlin, Germany Walter De Gruyter and Co. (1929).

[16] Beards, C.F. Structural vibration: analysis and damping. University of London, First published in Great Britain by Arnold (Hodder Headline Group) 276 (1996).

[17] Young, W.C., Budynas, R.G. Roark’s Formulas for Stress and Strain. (McGraw-Hill, 1989).

[18] Saeedi K, Bhat RB (2011). Clustered natural frequencies in multi-span beams with constrained characteristic functions. Shock. Vib..

[19] Caresta, M. Vibrations of a free–free beam. http://www.varg.unsw.edu.au/Assets/link%20pdfs/Beam_vibration.pdf

[20] Doyle, J.F. Flexural waves in beams, in Wave Propagation in Structures. (Springer, New York, 1989).

[21] Lin YK (1962). Free vibrations of a continuous beam on elastic supports. Int. J. Mech. Sci..

[22] Tao, Y. Impacts of various boundary conditions on beam vibrations. Graduate Theses, Dissertations, and Problem Reports 6774, West Virginia University (USA) (2015).

[23] Huang TC (1961). The effects of rotary inertia and of shear deformation on the frequency and normal mode equations of uniform beams with simple end conditions. J. Appl. Mech..

[24] Mead DJ, Yaman Y (1990). The harmonic response of uniform beams on multiple linear supports: a flexural wave analysis. J. Sound Vib..

[25] Chan HA, Paik HJ (1987). Superconducting gravity gradiometer for sensitive gravity measurements I.. Theory. Phys. Rev. D.

[26] Cleland AN, Roukes ML (2002). Noise processes in nanomechanical resonators. J. Appl. Phys..

[27] Saulson PR (1990). Thermal noise in mechanical experiments. Phys. Rev. D.

[28] Wang XQ, So RMC (2015). Timoshenko beam theory: a perspective based on the wave-mechanics approach. Wave Motion.

[29] Carrera, E., Giunta, G., Petrolo, M. Beam structures: classical and advanced theories. 208 (John Wiley and Sons Ltd, 2011).

[30] Philbin TG, Anders J (2016). Thermal energies of classical and quantum damped oscillators coupled to reservoirs. J. Phys. A Math. Theor..

[31] Degen CL, Reinhard F, Cappeliaro P (2017). Quantum sensing. Rev. Mod. Phys..

[32] Ganta D, Dale EB, Rezac JP, Rosenberger AT (2011). Optical method for measuring thermal accommodation coefficients using a whispering-gallery microresonator. J. Chem. Phys..

[33] Huang X, Deng Z, Xie Y, Fan J, Hu C, Tu L (2018). Study on misalignment angle compensation during scale factor matching for two pairs of accelerometers in a gravity gradient instrument. Sensors.

[34] Yan S, Xie Y, Zhang M, Deng Z, Tu L (2018). A subnano-g electrostatic force-rebalanced flexure accelerometer for gravity gradient instruments. Sensors.

[35] Chen Y (1963). On the vibration of beams or rods carrying a concentrated mass. J. Appl. Mech..

[36] Liu X, Du D, Mourou G (1997). Laser ablation and micromachining with ultrashort laser pulses. IEEE J. Quantum Electron..

