# Supplementary material for: Static and dynamic analyses of free-hinged-hinged-hinged-free beam in non-homogeneous gravitational field: application to gravity gradiometry
Source: Sci Rep. 2022 May 4;12:7215. doi: 10.1038/s41598-022-11232-6 (PMC9068812; doi:10.1038/s41598-022-11232-6)
Supplement: Supplementary file 1 — Supplementary Information. [file 41598_2022_11232_MOESM1_ESM.docx]

**Supplementary Materials**

The modal mirror-symmetric eigenfunctions for the free-hinged-hinged-hinged-free beam:

$$l\leq x\leq L$$

$$\psi_{n}^{\left( - \right)}\left( x \right)=\frac{\left( -1 \right)^{n}n\pi\left( L-l \right)\left( \mathrm{Cosh}\left[ \xi_{\left( - \right),n} \right]\mathrm{Sin}\left[ \xi_{\left( - \right),n}\frac{x-l}{L-l} \right]+\mathrm{Cos}\left[ \xi_{\left( - \right),n} \right]\mathrm{Sinh}\left[ \xi_{\left( - \right),n}\frac{x-l}{L-l} \right] \right)}{\xi_{\left( - \right),n}l\left( \mathrm{Cos}\left[ \xi_{\left( - \right),n} \right]+\mathrm{Cosh}\left[ \xi_{\left( - \right),n} \right] \right)}$$

$$-l\leq x\leq l$$

$$\psi_{n}^{\left( - \right)}\left( x \right)=\mathrm{Sin}\left[ \pi n\left( x/l \right) \right]$$

$$-L\leq x\leq-l$$

$$\psi_{n}^{\left( - \right)}\left( x \right)=\frac{\left( -1 \right)^{n}n\pi\left( L-l \right)\left( \mathrm{Cosh}\left[ \xi_{\left( - \right),n} \right]\mathrm{Sin}\left[ \xi_{\left( - \right),n}\frac{x+l}{L-l} \right]+\mathrm{Cos}\left[ \xi_{\left( - \right),n} \right]\mathrm{Sinh}\left[ \xi_{\left( - \right),n}\frac{x+l}{L-l} \right] \right)}{\xi_{\left( - \right),n}l\left( \mathrm{Cos}\left[ \xi_{\left( - \right),n} \right]+\mathrm{Cosh}\left[ \xi_{\left( - \right),n} \right] \right)}$$

where $\xi_{\left( - \right),n}$ approximates as follows

$$\xi_{\left( - \right),n}\cong\frac{{10}^{-8}}{n^{8}}+ Tanh\left[ 1.5778901\pi\left( n - 1 \right) \right]\left( \frac{\pi}{4}+ \pi\left( n - 1 \right) \right)$$

$$n=1,2,3\ldots$$

The modal symmetric eigenfunctions for the free-hinged-hinged-hinged-free beam:

$$l\leq x\leq L$$

$$\psi_{n}^{\left( + \right)}\left( x \right)=\frac{(L-l)(1-\mathrm{Csch}[\xi_{\left( + \right),n}]\mathrm{Sin}[\xi_{\left( + \right),n}])(\mathrm{Sec}[\xi_{\left( + \right),n}]\mathrm{Sin}[\xi_{\left( + \right),n}\frac{x-l}{L-l}]+\mathrm{Sech}[\xi_{\left( + \right),n}]\mathrm{Sinh}[\xi_{\left( + \right),n}\frac{x-l}{L-l}])}{l(1+\mathrm{Cos}[\xi_{\left( + \right),n}]\mathrm{Sech}[\xi_{\left( + \right),n}])}$$

$$0\leq x\leq l$$

$$\psi_{n}^{\left( + \right)}\left( x \right)=\left( \mathrm{Cosh}[\xi_{\left( + \right),n}\frac{x}{l}]-\mathrm{Coth}[\xi_{\left( + \right),n}]\mathrm{Sinh}[\xi_{\left( + \right),n}\frac{x}{l}] \right)\mathrm{Tan}[\xi_{\left( + \right),n}]-\mathrm{Sec}[\xi_{\left( + \right),n}]\mathrm{Sin}[\xi_{\left( + \right),n}\frac{l-x}{l}]$$

$$-l\leq x\leq0$$

$$\psi_{n}^{\left( + \right)}\left( x \right)=\left( \mathrm{Cosh}\left[ \xi_{\left( + \right),n}\frac{x}{l} \right]+\mathrm{Coth}[\xi_{\left( + \right),n}]\mathrm{Sinh}[\xi_{\left( + \right),n}\frac{x}{l}] \right)\mathrm{Tan}[\xi_{\left( + \right),n}]-\mathrm{Sec}[\xi_{\left( + \right),n}]\mathrm{Sin}[\xi_{\left( + \right),n}\frac{l+x}{l}]$$

$$-L\leq x\leq-l$$

$$\psi_{n}^{\left( + \right)}\left( x \right)=-\frac{(L-l)(1-\mathrm{Csch}[\xi_{\left( + \right),n}]\mathrm{Sin}[\xi_{\left( + \right),n}])(\mathrm{Sec}[\xi_{\left( + \right),n}]\mathrm{Sin}[\xi_{\left( + \right),n}\frac{x+l}{L-l}]+\mathrm{Sech}[\xi_{\left( + \right),n}]\mathrm{Sinh}[\xi_{\left( + \right),n}\frac{x+l}{L-l}])}{l(1+\mathrm{Cos}[\xi_{\left( + \right),n}]\mathrm{Sech}[\xi_{\left( + \right),n}])}$$

where $\xi_{\left( + \right),n}$ approximates as follows

$$\xi_{\left( + \right),n}=Tanh[1.5778901\pi n](\frac{\pi}{4}+\pi n)$$

$$n=1,2,3\ldots$$

All modal eigenfunctions above can be arbitrary normalised by the following transfer

$$\psi_{n}^{(\pm)}\left( x \right)\to\frac{N}{\sqrt{\int_{-L}^{L} dx\psi_{n}^{(\pm)}\left( x \right)\psi_{n}^{(\pm)}\left( x \right)}}\psi_{n}^{(\pm)}\left( x \right)$$

where *N* is a normalisation factor (chosen to be $\sqrt{2L}$ throughout this paper).

The modal eigenvalues $k_{\left( \pm\right),n}$ for the whole beam’s length are found from Eq.34 as follows

$$k_{\left( \pm\right),n}=\left( \frac{\int_{-L}^{L} dx\psi_{n}^{(\pm)}\left( x \right)\frac{d^{4}}{{dx}^{4}}\psi_{n}^{(\pm)}\left( x \right)}{\int_{-L}^{L} dx\psi_{n}^{(\pm)}\left( x \right)\psi_{n}^{(\pm)}\left( x \right)} \right)^{1/4}$$
